# Tailoring Healthy Workplace Interventions to Local Healthcare Settings: A Complexity Theory-Informed Workplace of Well-Being Framework

**DOI:** 10.1155/2015/340820

**Published:** 2015-08-25

**Authors:** Sarah L. Brand, Lora E. Fleming, Katrina M. Wyatt

**Affiliations:** ^1^Plymouth University Peninsula Schools of Medicine and Dentistry, Plymouth PL6 8BX, UK; ^2^European Centre for Environment and Human Health, University of Exeter Medical School, Truro TR1 3HD, UK; ^3^University of Exeter Medical School, Exeter EX2 4SG, UK

## Abstract

Many healthy workplace interventions have been developed for healthcare settings to address the consistently low scores of healthcare professionals on assessments of mental and physical well-being. Complex healthcare settings present challenges for the scale-up and spread of successful interventions from one setting to another. Despite general agreement regarding the importance of the local setting in affecting intervention success across different settings, there is no consensus on what it is about a local setting that needs to be taken into account to design healthy workplace interventions appropriate for different local settings. Complexity theory principles were used to understand a workplace as a complex adaptive system and to create a framework of eight domains (system characteristics) that affect the emergence of system-level behaviour. This Workplace of Well-being (WoW) framework is responsive and adaptive to local settings and allows a shared understanding of the enablers and barriers to behaviour change by capturing local information for each of the eight domains. We use the results of applying the WoW framework to one workplace, a UK National Health Service ward, to describe the utility of this approach in informing design of setting-appropriate healthy workplace interventions that create workplaces conducive to healthy behaviour change.

## 1. Introduction

Developing a complex intervention to affect healthy behaviour change in healthcare settings is subject to numerous challenges related to setting complexity, in particular difficulties in the “scale-up” and spread of a successful intervention in one setting to a different setting [1]. There is an emerging trend in health services research to acknowledge and consider the importance of local context as a major factor in how a particular intervention will be played out in that setting and whether or not a successful intervention in another setting can be transferred to a new setting. Furthermore, there is a call within social science for complex interventions, which have multiple, synergistic components and interact with context [2] to be properly theorised [3, 4] (e.g., [5–8]).

However, there is no consensus in the literature regarding how to choose which information to gather from a setting to inform setting-appropriate intervention development or indeed which aspects of a setting may affect the success of a particular intervention. The World Health Organisation (WHO), in its healthy workplace framework, describes an assessment of the present situation of the workplace as one stage of developing a healthy workplace intervention [9], but there is no mention of the dynamics of the workplace system and how these interact with the current situation in each setting to impact the outcome of an intervention.

Complexity theory [10–12] offers a theoretical framework to support intervention design and implementation that acknowledges and works with the complexity of the setting in which the intervention will be put into practice. Complexity science principles are increasingly being used for understanding system-level behaviour and organisational change in complex settings [13–15], including healthcare organisations [16–23].

Healthcare settings are considered to be “complex adaptive systems” because they are made up of groups of individual agents who are free to act in ways that are not entirely predictable and whose actions are interconnected such that one individual's action will change the context for the other agents in the system [17]. All of the elements of a complex adaptive system that affect the system-level behaviour are interrelated and are coevolving, and thus patterns of behaviour in the system are unpredictable and unlikely to be altered in a stepwise fashion. These types of systems are also open systems, in that they are a component of a wider system, adding to the number of influences on the effect of an intervention in a given setting.

Complexity theory also offers a promising perspective from which to understand the interactions between local context and intervention outcomes. It highlights the dynamic and relational properties of a particular setting and those aspects that enable the people in it to organise themselves into new ways of working, thinking, and relating. For complex healthcare settings, this means both understanding and working with and within the environmental and relational characteristics of the system [24]. Patterns of health-related behaviour are seen as an emergent property of the workplace system: changing the behaviour of people in the healthcare setting (e.g., nurses, doctors, and administrative staff) is unlikely to be achieved through external or top-down input alone or by targeting particular behaviours or at-risk groups in isolation from their context [12, 25]. New behaviours should be facilitated by affecting the dynamic relational properties and physical environment of the workplace system.

Whilst system behaviour at any point in time is hard to predict, patterns of behaviour in complex adaptive systems are able to be seen over time, for example, weather patterns over the seasons [26]. Certain aspects of complex systems are seen to influence patterns of behaviour emerging in that system, and thus patterns of behaviour in a given system will emerge from the unique conditions of that system at that time in relation to these aspects of the system. In workplace settings, these include the rules, beliefs, and values that the people making up the system share, along with the nature of the interactions between the agents in the system over time [12, 14, 23].

In this paper, we use the principles of complexity theory to design a framework to guide the development and the implementation of setting-appropriate intervention activities. We describe applying the Workplace of Well-being (WoW) framework to one NHS hospital ward to illustrate the value of this approach in gaining a rich understanding of a setting in terms of its local system dynamics and relational aspects that affect system-level behaviour in order to support context-appropriate development of healthy workplace activities and processes.

## 2. Materials and Methods

First, we describe the development of the WoW framework from complexity principles and briefly introduce the method and thematic results of the NHS case study (reported in detail elsewhere [27]); then using findings from this case study we describe how the application of the WoW framework to a local workplace leads to an understanding of its local characteristics and finally how the WoW framework could guide the design of healthy workplace interventions which are appropriate for the unique system dynamics, culture, context, and relational aspects of a particular workplace.

### 2.1. Development of the Framework

Reviewing the literature regarding complexity theory in the social sciences and in particular in regard to healthcare settings, we considered how complexity theory could inform the development of an intervention which would be sensitive to and appropriate for the unique nature of the individual hospital ward setting.  Table 1 describes the aspects of complexity theory considered and how we have conceptualised them in relation to creating healthy workplaces.

**Figure 1:**
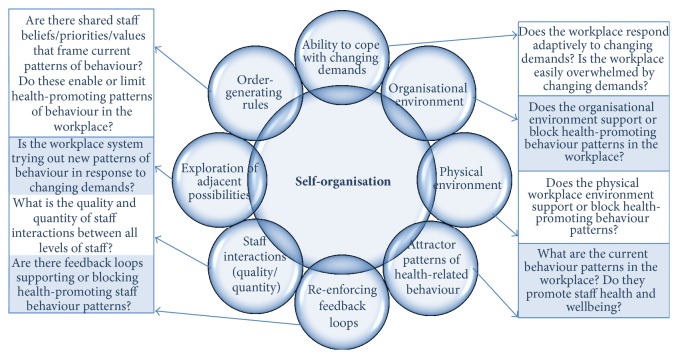
The Workplace of Well-being (WoW) framework developed from the principles of complex adaptive system theory guides exploration of eight interrelated workplace characteristics contributing to the ability of a workplace system to self-organise into more health-promoting patterns of behavior.

From the complex adaptive system principles described in Table 1, we identified eight interrelated domains that may impact on a workplace's ability to self-organise into new patterns of behaviour over time. The eight domains are aspects of the workplace that should be considered when seeking to understand the unique nature of a particular workplace to aid setting-appropriate and setting-sensitive intervention development. The WoW framework guides the understanding of aspects of the interrelated context, culture, and dynamic nature of the local setting that enables or blocks the dynamical ability of the system to self-organise into new patterns of behaviour. The eight interrelated domains in the Workplace of Well-being (WoW) framework are illustrated in Figure 1.

Using the WoW framework, a shared understanding of the relationships and behaviours within a workplace is cocreated which allows an understanding of its characteristics as a complex system (e.g., how information flows through the system or how patterns of behaviour form and evolve), as well as specific contextual information (e.g., the local patterns of behaviour in the system at that particular time that will frame which new patterns of behaviour are possible given current conditions).

To apply the WoW framework to a workplace, data collection is carried out to assess the current conditions in that workplace guided by the eight domains in WoW (Figure 1). Figure 1 shows the overarching questions to be asked of the workplace for each domain. Thematic analysis within each domain then results in a detailed description of the workplace in terms of that complex adaptive system characteristic and the enablers and barriers in that domain to self-organisation into new patterns of workplace behaviour. This understanding then supports design and implementation of intervention activities appropriate to that unique workplace system.

### 2.2. Method and Thematic Results from NHS Ward Case Study [27]

The WoW framework was applied to a single NHS ward within acute healthcare trust to guide the development of appropriate intervention activities to improve staff health and well-being. Potential wards were identified in close collaboration with the senior management team of an NHS acute hospital. A convenience sampling approach identified one ward and all permanent ward staff in that ward were invited to participate in individual open-ended interviews where they were asked to reflect on structural and behavioural barriers and facilitators to their health and well-being at their workplaces and the nature of relations within the ward and with the wider hospital trust. Data was analysed thematically [28]. The interviewer and one of two other members of the research team double-coded the interviews independently and discussed relevant emerging themes. Codes were developed to inductively classify data within each theme. Developing summary themes or categories from raw transcripts in this way enables an understanding of the meaning present in the complex data [29]. All staff were invited to group workshops to feed back the themes and facilitate a shared understanding of the local conditions of their workplace.

Themes developed from the staff interviews were hierarchy of care, unpredictable workload, environmental barriers to health and well-being, break-taking, and relationships [27].

## 3. Results

We now describe how the application of the WoW framework to a local workplace leads to an understanding of its local characteristics in complexity terms, based on the thematic results from the NHS case study. First we describe the enablers and barriers to system change in relation to each of the eight domains of the WoW framework. We then show how these findings can be translated to design the kind of intervention that would be appropriate to this particular workplace.

### 3.1. Ability to Cope with Changing Demands

The workload on the ward was described as unpredictable and changeable with a clear sense of lack of control and uncertainty as to what the workload would be on each shift and a feeling of not having the personal resources (time/energy) to meet the demands of the job. Whilst demands were constantly changing, ward staff's experiences suggest that the ward, at the system-level, was unable to appropriately adapt its behaviour in response.

### 3.2. Organisational Environment

The ward seemed to represent a microcosm of the wider hospital environment. The organisational environment surrounding the ward was experienced as uncaring, unapproachable, and governed by inflexible rules and regulations that did not reflect the hopes and beliefs of staff.

### 3.3. Physical Environment

The physical ward environment directly limited exploration of more health-promoting behaviours, for example, the lack of a suitable break-taking space for taking proper breaks. Moreover, the physical ward issues of the staff room and windows which had been previously highlighted to off-ward management and the lack of management response to complaints seemed to encapsulate for staff their feeling of being unheard and uncared for in the organisation; this further fed into the shared belief that management were not on their side and the corresponding “us versus them” pattern of behaviour.

### 3.4. Attractor Patterns of Health-Related Behaviour

Patterns of behaviour on the ward that appeared to limit staff health and well-being included non-engagement in self-care (i.e., hierarchy of care was patients, then other staff, and finally staff themselves), limited break-taking, and an “us versus them” culture between frontline staff and management level staff.

### 3.5. Exploration of Adjacent Possibilities

These behaviour patterns had become entrenched, and there was a feeling amongst ward staff that this was just the way things were on this ward. Historically, staff felt there was a lack of response from management when raising work-related concerns, and getting change to happen through contact with off-ward management was described as “like pulling teeth.” For example, staff expressed frustration and stress related to the physical ward environment and their powerlessness to bring about desired changes to it. This related particularly to the loss of their staff room and not being allowed to open the windows on the ward even in summer (due to patient safety and hospital liability concerns).

This perceived lack of response from management led to a lack of belief in the possibility of change and reluctance to try out new behaviours. In complexity terms, this is “path dependency” and describes the “locked-in behaviour” in the ward, with the system unable to create and explore new possible behaviours.

### 3.6. Re-Enforcing Feedback Loops

These “locked-in” behaviours were supported by several re-enforcing feedback loops. For example, the “us versus them” pattern of behaviour between the wider management and ward staff described a situation in which the beliefs and behaviours of the two groups and the lack of relations between them appeared to have become systemically embedded. This pattern also appeared to feed into the ward staff beliefs that management were not on their side and that change was not possible.

### 3.7. Ward Staff Interactions (Quality/Quantity)

Staff felt a strong sense of team spirit on the ward which was felt to be supportive to their mental well-being. In contrast, a strong “us versus them” culture reflected by a perceived lack of communication, trust, and shared understanding with off-ward management staff underlies many of the descriptions of stress and frustration for ward staff.

### 3.8. Order-Generating Rules

Three particular order-generating rules or shared staff beliefs/priorities/values were identified that appeared to frame patterns of health-related behaviour on the ward: (1) patient care and team care supersede self-care; (2) change is not possible; and (3) management are not on our side. These rules supported the locked-in behaviour of the ward, attracting staff behaviour towards limited break-taking; low engagement in self-care activities at work; and low quality and quantity interactions with management staff.

### 3.9. What Kinds of Interventions Are Appropriate for This Ward Environment?

In a complex adaptive system, system-level behaviour change is possible when the system is able to explore new ways of behaving. Supporting the ward's propensity to self-organise into new behaviours would support the ward to adaptively respond to its changing environment with appropriate new ways of behaving, without becoming overwhelmed by change. This would both directly reduce stressors on ward staff (e.g., feeling unable to cope with an unpredictable overwhelming workload) and allow for the emergence of new and healthier patterns of staff behaviour on the ward (e.g., healthy break-taking and an inclusive, compared to “us versus them,” ward culture).

The understanding of this particular ward's enablers and barriers to its self-organising into new ways of behaving highlights how to create the conditions in the ward that would enable more health-promoting behaviours to emerge. Healthy workplace interventions in this ward would need to take into account the factors that are limiting or enabling the ward system's dynamics and consider how an incoming intervention would interact with the current dynamic properties of this ward system.

Introducing a healthy workplace intervention will not produce sustainable behaviour change in the workplace if it does not work to enable the ability of the workplace to self-organise into new patterns of behaviour. In this particular ward, an intervention would need to target the “us versus them” culture and the feedback loops that support it, support better quality and quantity of communication between parts of the ward system, address the physical environment issues that limit break-taking ability and that support feelings of being unheard and uncared for in the organization, and take into consideration the social rules that create the current patterns of ward behaviour. In these ways, an intervention could enable the self-organisation of the system to support staff behaviour to change at the system-level and thus produce a ward environment that is conducive to healthy behaviour change in the intervention.

Activities (such as regular healthy workplace meetings with staff and management) that provide opportunities for new and more diverse relations to be built between different levels of ward staff across the perceived “us versus them” boundaries could increase the quality and quantity of interactions between ward and management staff in turn altering the “us versus them” pattern of behaviour and supporting the self-organisational dynamics of the system towards more healthful behaviours.

Creating “small wins” (i.e., bringing about even small positive desired changes) for this ward could be fundamental in changing their shared belief in the lack of possibility for change and supporting the testing out of new ways of behaving or, in complexity terms, the exploration of the space of possibilities. For this ward, potential small wins are available in the form of changes to the physical ward environment (e.g., windows that open). The complex interrelations in a complex adaptive system mean that small inputs such as these have the potential to bring about nonproportionate (e.g., small input = large output) distributed change throughout the system.

Ward staff learning through small wins requires positive feedback loops that feed the benefits of the small win back into the system to influence staff beliefs and support the emergence of new behaviours. Current feedback loops in the system seemed to act to support the ward not trying out new behaviours, perhaps because these loops were “local” and nondiverse. Interventions that enable or involve the (self-) identification of workplace champions who facilitate interactions between the ward and management would increase the diversity of information and exposure to new ideas and ways of doing things on the ward, as well as creating new feedback loops which could support new ways of behaving.

Similarly, interventions enabling the recognition and addressing of ward staff voiced needs by management through small wins could allow a new type of relation and interaction between ward staff and management to form. These small wins have the potential to “unlock” staff behaviour patterns through feedback loops that change staff beliefs and create transformative behaviour change in the ward system [25].

In complex adaptive social systems, the patterns of behaviour that can emerge are framed by the order-generating rules that the people in the system share [30]. Shared beliefs and priorities on this ward framed some of its health-limiting patterns of behaviour. Interventions that support staff to consider their beliefs, behaviours, and how they are related in facilitated workshops could begin to change staff beliefs and allow the exploration of possible new healthier behaviours [31]. Similarly workshops with management which allow staff to express their beliefs could increase lines of communication and allow for issues to be resolved mutually.

To ensure that there is a wider receptive context with which the “nested” ward system can coevolve, it is critical that interventions have the active long-term support and understanding of the hospital senior management team and board.

## 4. Discussion

We describe the development and implementation of the Workplace of Well-being (WoW) framework. The WoW framework was conceptualised from our understanding of the workplace as a complex adaptive system, in order to elucidate what it is about a workplace that is important in determining the success of an intervention to support healthy behaviour change. The WoW framework describes eight domains that are important to consider about a workplace when designing healthy behaviour change interventions. This framework can be applied to individual settings to gather detailed local data about the enablers and barriers regarding the workplace's ability to self-organise into new and sustainable behaviours. We describe how the framework, when applied to one NHS ward [27], informs what kind of intervention activities are appropriate, feasible, and acceptable for that unique workplace.

### 4.1. Supporting Setting-Appropriate Intervention Activities

Using the complexity-informed WoW framework an understanding of the characteristics of a particular workplace setting that are enablers or barriers to system-level behaviour change can be gained. This informs which aspects of the workplace need to be targeted by a healthy workplace intervention to support the adoption of more healthful behaviours. In the case of the NHS ward, this means opportunities for increased quality, quantity, and diversity of relations and interactions between ward and management staff, small quick wins to encourage shared belief in the possibility and the benefits of change, facilitation of re-enforcing feedback loops to support trying new behaviours, and facilitation of action learning to explore health-limiting shared beliefs.

### 4.2. Creating a Change-Conducive Setting

Using the WoW framework, the setting-specific enablers and barriers to intervention implementation can also be identified and addressed as part of the intervention. In the NHS case study, the WoW framework identified a “locked-in” behaviour pattern on the ward. For this workplace, an intervention implemented without first, or at the same time, enabling the system to self-organise would not be likely to support new ways of behaving/working. According to the principles of complexity theory, if an intervention includes elements that create the conditions for self-organisation, then it will be more likely to lead to sustainable behaviour change [34]. An intervention will be unlikely to affect change if the barriers to behaviour change in a particular system are not addressed.

### 4.3. Why Local Context Is Important: A Complexity Perspective

Thinking of complex settings in terms of complex adaptive system characteristics highlights why the local context is so important in the success of interventions in complex settings; complex settings have complex dynamic and relational properties that mean the system does not behave in a linear and predictable fashion. Predicting the effect of external inputs to the system, such as an intervention added to a system, is difficult. This is because the intervention itself (as well as any researchers or practitioners who “join” the system as part of its implementation) will interact with the system in a myriad of ways. Targeting interventions such that they take into account and work with or support the dynamic, relational character of the system offers a new way of conceptualising interventions as part of a living, evolving system.

### 4.4. Redefining “Best Practice” When Transferring Interventions to New Complex Settings

The WoW framework supports the development of interventions that address the problem of scale-up and spread of interventions in healthcare settings [1]. In the “best practice” culture in the UK health service, interventions found to have a positive effect in one setting are implemented in as close to exactly the same way as possible in another setting, with the result that many fail to have a positive impact in the new setting [1].

From a complexity perspective, a behaviour that emerges in one setting is particular to the history, dynamics, and relational properties of that setting and the way those unique properties interact with an intervention. Taking an intervention that “produces” a particular system-level behaviour in one unique setting and scaling it up to fit the wider setting (“scale-up”) or transplanting it to another unique setting (“spread”) is unlikely to produce the same behavioural outcome.

The WoW framework guides an understanding of the history, dynamics, and relational properties of a healthcare setting. Developing interventions using this framework at a midlevel of abstraction (i.e., a description of the properties shared by social complex adaptive systems) allows scale-up and spread of the intervention framework rather than the intervention activities themselves [1]. Specific intervention activities that are appropriate to each individual setting can then be developed by populating the framework with local information. In this way, the transfer of best practice becomes the transfer of the midlevel framework of how a social complex adaptive system works and what, in a more abstract sense, its properties are. Intervention development then becomes the development of local activities that target the unique enablers and barriers in that particular setting which have been identified by the individuals located in the setting.

### 4.5. Sustainability of Interventions in Complex Settings

Designing intervention activities that take into account both the dynamic properties of the system and its particular history and space of possibilities (i.e., its possible adjacent patterns of behaviour based on its current properties, dynamics, behaviour patterns, and so on; see Table 1) creates interventions designed to change the way a system behaves. The intervention activities described in relation to the NHS case study, which target the self-organising dynamics of the system itself, can become a sustainable part of the workplace system because they affect the very way the system behaves. For example, by enabling feedback loops that support the evolution of system behaviour in response to its environment, the WoW framework has the potential to create sustainable interventions that evolve with the system they are a part of.

## 5. Limitations

We sought to apply complexity science as a theoretical framework to consider how to affect the system's properties (in this case, a ward in a hospital) towards health-promoting behaviours. However, whether this approach can deliver such change needs to be properly evaluated in future studies. We also acknowledge that the process of applying the WoW framework to a workplace will require additional time and resources both from the workplace and the researchers, compared to implementing “one-size-fits-all” interventions. This kind of investment in better preparing interventions for their intended particular local contexts should pay off in terms of better outcomes in the short and long term; nevertheless this payoff needs to be explored in future trials of the WoW framework in different workplaces.

## 6. Conclusions

The WoW framework has the potential to be a useful tool to support the development of setting-appropriate healthy workplace interventions in complex healthcare settings. This framework supports data collection from local settings and guides analysis to understand the local dynamics and relational properties of complex social systems. Thus, the WoW framework can inform the development of intervention activities likely to support local workplace behaviour change. Intervention activities developed in this way are led by local needs, are setting-appropriate, and are supported by an understanding of how the local setting can be enabled to support the implementation and sustainability of the intervention.

The WoW framework offered a constructive perspective from which to consider the health-related behaviours of the ward staff on one NHS ward as well as the multiple system-level factors that they are emergent products of. The framework helps to identify the kinds of intervention activities that would be appropriate to sustainably support health-promoting staff behaviour change in this workplace.

Interventions developed using the WoW framework have good potential for scale-up and spread across diverse settings, a challenge in current best practice transfer in healthcare [1], because the framework and its implementation are transferrable across different healthcare settings and the resultant intervention activities setting-specific. Nevertheless, further work is needed to explore the feasibility and acceptability of the WoW framework to staff in other healthcare settings and to examine the long-term effectiveness of setting-specific intervention activities developed using this framework.

## Figures and Tables

**Table 1:** The principles of complex adaptive systems (CAS) and how each principle is relevant to developing setting-appropriate interventions in workplace systems.

CAS principle	Relevance for workplace setting-appropriate intervention development
Interrelatedness and distributed control	All elements of a complex adaptive system are interrelated and are coevolving and behaviour change in complex healthcare settings is an emergent property of the interrelated and complex interactions between different workplace elements. Control is not centralised and top-down [12] and nor is it bottom-up, with power being something that can be “given” to the agents of a system [25]. Interventions cannot empower local agents (e.g., staff) by handing them the power to change their work environment; empowerment must take the form of enabling change to emerge within their context.

Order-generating rules	Patterns of behaviour in complex adaptive systems emerge from the operation of a few simple order-generating rules [14, 30]. Order-generating rules in a workplace include staff's shared instincts, values, priorities, constructs, and mental models, for example, in a healthcare setting, the internalised rule of “first, do no harm” [23].

Edge of chaos	The edge of chaos is a point between chaos and order where a complex adaptive system has the most creativity, growth, and ability to adaptively change; it neither settles into stable equilibrium, nor quite falls apart [11, 32]. If a workplace is too stable, nothing changes; if it is too chaotic, the workplace will be overwhelmed by change. In either case, the workplace will be unable to adaptively change to its changing environment unless new order-generating rules are established [33] that act to hold the system at the edge of chaos [30]. Interventions to change behaviour in these systems need to focus on the enablers and barriers for the system to continuously self-organise into adaptive ways of behaving in response to its changing environment, for example, by enabling the dynamics of the workplace system (e.g., increasing interaction quality and quantity between staff) such that the system can continuously adapt to its environment and establish new order-generating rules and thus be neither too chaotic nor too ordered.

Self-organisation	Self-organisation refers to the internal propensity of complex adaptive systems toward more organised patterned behaviour [10, 11]. System-level patterns of behaviour emerge *without external input or central control*. This patterned behaviour is *emergent*, emerging from the interactions and relations of the interdependent agents (i.e., staff) in the system. These interactions are constrained and guided by the implicit or explicit order-generating rules (or shared priorities and values) of the staff. Interventions to change behaviour in a complex social system should enable the self-organising dynamics to support both sustainable adaptive change in the system and the integration of the intervention into the way that the system works.

Attractor patterns	Attractor patterns are patterns of behaviour that a complex adaptive system is attracted toward because of its particular conditions [11]. Staff behaviour patterns are those staff are drawn towards behaving in by the particular conditions of the workplace system at that time (i.e., the interrelations between staff and the order-generating rules or shared values they hold): changing these conditions will attract staff toward different patterns of behaviour [18]. Understanding how people in a setting are drawn towards behaving in certain ways shows how changing the underlying context can draw people to behave in different ways.

Re-enforcing feedback loops	Patterns emerging from the interactions of agents (e.g., staff) feed back into the system and further influence the shared beliefs and interactions of the agents. Feedback loops support the continuation of particular patterns of behaviour through the local experience of agents (e.g., staff) and can support the adaptive dynamic behaviour change in a system in response to its environment [25].

Coevolution of system and its environment	A complex adaptive system has the ability to continually create new order *in coevolution with its environment *[24]. This involves not only continual adaptation *to* its environment (i.e., the workplace's wider social, cultural, and physical environment), but also the influencing *of* its environment through its changed behaviour. Interventions at a local level can create distributed change throughout the wider system that the local setting is a part of, and the wider system will continuously affect the local setting. Awareness of the wider context, for example, the organisational culture when looking at an individual team or department, is important to support appropriate intervention development and also an understanding of potential barriers or enablers to intervention implementation (e.g., management level support, or not, for a local intervention).

Sensitivity to initial conditions	The characteristics of a social complex adaptive system are highly context specific, not responding in the same way to the same stimulus under different circumstances at different times [12, 15]. System change will always begin from and involve the evolution of the initial conditions present in the system at the time. Behaviour change in a particular workplace can only begin from the particular context of that workplace and thus it is crucial that interventions are tailored to local workplaces' initial conditions.

Creation of adjacent possibilities and awareness of path dependency	The space of possibilities for a complex adaptive system includes all of the possible (adjacent) new patterns of behaviour available at that time, *given the initial conditions* of the system. Initial conditions determine the adjacent possible patterns of behaviour, leading to “path dependency”: a system's current behaviour is dependent upon its history; its previous behaviour made its current behaviour possible, which determines its possible next behaviours (adjacent possibilities). System-level behaviour change emerges from the exploration of new adjacent patterns of behaviour by a system [10, 30]. Depending on its particular dynamics each workplace will have a different space of possibilities and will differ in its ability to test out new ways of behaving. An understanding of the current conditions of a complex setting will help intervention developers to design an intervention that is appropriate for that workplace and that aims to bring about changes that are feasible given the characteristics of that workplace.

## References

[1] Lanham H. J., Leykum L. K., Taylor B. S., McCannon C. J., Lindberg C., Lester R. T. (2013). How complexity science can inform scale-up and spread in health care: understanding the role of self-organization in variation across local contexts. *Social Science & Medicine*.

[2] Craig P., Dieppe P., Macintyre S. (2008). *Developing and Evaluating Complex Interventions*.

[3] Bonell C., Fletcher A., Morton M., Lorenc T., Moore L. (2012). Realist randomised controlled trials: a new approach to evaluating complex public health interventions. *Social Science & Medicine*.

[4] Moore G., Audrey A., Barker M. (2014). *Process Evaluation of Complex Interventions: UK Medical Research Council (MRC) Guidance*.

[5] Foy R., Francis J. J., Johnston M. (2007). The development of a theory-based intervention to promote appropriate disclosure of a diagnosis of dementia. *BMC Health Services Research*.

[6] French S. D., Green S. E., O'Connor D. A. (2012). Developing theory-informed behaviour change interventions to implement evidence into practice: a systematic approach using the Theoretical Domains Framework. *Implementation Science*.

[7] Glidewell L., Boocock S., Pine K. (2013). Using behavioural theories to optimise shared haemodialysis care: a qualitative intervention development study of patient and professional experience. *Implementation Science*.

[8] Hrisos S., Eccles M., Johnston M. (2008). Developing the content of two behavioural interventions: using theory-based interventions to promote GP management of upper respiratory tract infection without prescribing antibiotics #1. *BMC Health Services Research*.

[9] Burton J., World Health Organization (2010). *WHO Healthy Workplace Framework and Model: Background and Supporting Literature and Practices*.

[10] Goodwin B. (1997). *How the Leopard Changed Its Spots*.

[11] Kauffman S. A. (1993). *The Origins of Order: Self Organization and Selection in Evolution*.

[12] Holland J. (1998). *Emergence: From Chaos to Order*.

[13] Mitleton-Kelly E. (2003). *Ten Principles of Complexity and Enabling Infrastructures*.

[14] Stacey R. D. (2003). *Strategic Management and Organisational Dynamics: The Challenge of Complexity*.

[15] Keshavarz N., Nutbeam D., Rowling L., Khavarpour F. (2010). Schools as social complex adaptive systems: a new way to understand the challenges of introducing the health promoting schools concept. *Social Science & Medicine*.

[16] Mitleton-Kelly E. (2011). A complexity theory approach to sustainability: a longitudinal study in two London NHS hospitals. *The Learning Organization*.

[17] Plsek P. E., Wilson T. (2001). Complexity science: complexity, leadership, and management in healthcare organisations. *British Medical Journal*.

[18] Plsek P. E., Greenhalgh T. (2001). Complexity science: the challenge of complexity in health care. *The British Medical Journal*.

[19] Begun J. W., Zimmerman B., Dooley K., Mick S. M., Wyttenbach M. (2003). Health care organizations as complex adaptive systems. *Advances in Health Care Organization Theory*.

[20] Litaker D., Tomolo A., Liberatore V., Stange K. C., Aron D. (2006). Using complexity theory to build interventions that improve health care delivery in primary care. *Journal of General Internal Medicine*.

[21] Rowe A., Hogarth A. (2005). Use of complex adaptive systems metaphor to achieve professional and organizational change. *Journal of Advanced Nursing*.

[22] Plsek P. E., The Committee on the Quality of Health Care in America (2001). Redesigning health care with insights from the science of complex adaptive systems. *Crossing the Quality Chasm: A New Health System for the 21st Century*.

[23] Plsek P. E. Complexity and the adoption of innovation in health care.

[24] Mitleton-Kelly E. (2011). Identifying the multi-dimensional problem-space and co-creating an enabling environment. *Emergence: Complexity & Organization*.

[25] Durie R., Wyatt K. (2007). New communities, new relations: the impact of community organization on health outcomes. *Social Science & Medicine*.

[26] Gell-Mann M. (1995). *The Quark and the Jaguar: Adventures in the Simple and the Complex*.

[27] Wyatt K. M., Brand S., Ashby-Pepper J., Abraham J., Fleming L. E. (2015). Understanding how healthy workplaces are created: implications for developing a national health service healthy workplace program. *International Journal of Health Services*.

[28] Braun V., Clarke V. (2006). Using thematic analysis in psychology. *Qualitative Research in Psychology*.

[29] Thomas D. R. (2006). A general inductive approach for analyzing qualitative evaluation data. *American Journal of Evaluation*.

[30] Burnes B. (2005). Complexity theories and organizational change. *International Journal of Management Reviews*.

[31] Kernick D. (2002). The demise of linearity in managing health services: a call for post normal health care. *Journal of Health Services Research & Policy*.

[32] Brown S. L., Eisenhardt K. M. (1997). The art of continuous change: linking complexity theory and time-paced evolution in relentlessly shifting organizations. *Administrative Science Quarterly*.

[33] MacIntosh R., MacLean D. (2001). Conditioned emergence: researching change and changing research. *International Journal of Operations & Production Management*.

[34] Durie R., Wyatt K. (2013). Connecting communities and complexity: a case study in creating the conditions for transformational change. *Critical Public Health*.

